# Retina-to-brain spreading of α-synuclein after intravitreal injection of preformed fibrils

**DOI:** 10.1186/s40478-023-01575-0

**Published:** 2023-05-20

**Authors:** Dayana Pérez-Acuña, Ka Hyun Rhee, Soo Jean Shin, Jeeyun Ahn, Jee-Young Lee, Seung-Jae Lee

**Affiliations:** 1 Department of Biomedical Sciences, Seoul National University College of Medicine, 103 Daehak-Ro, Jongro-Gu, Seoul, 03080 Korea; 2 Department of Ophthalmology, College of Medicine, Seoul Metropolitan Government-Seoul National University Boramae Medical Center, Seoul National University, Seoul, South Korea; 3 Department of Neurology, Seoul Metropolitan Government-Seoul National University Boramae Medical Center, Seoul National University College of Medicine, Seoul, South Korea; 4 Neuroscience Research Institute, Seoul National University College of Medicine, Seoul, South Korea; 5 Convergence Research Center for Dementia, Seoul National University College of Medicine, Seoul, South Korea; 6 Neuramedy, Seoul, South Korea; 7 Present Address: Department of Biochemistry, Molecular Biology and Biophysics, University of Minnesota, Minneapolis, MN 55455 USA

**Keywords:** Parkinson’s disease, α-synuclein, Protein aggregation, Retina, Retinal degeneration

## Abstract

Parkinson’s disease (PD) is a neurodegenerative disorder characterized by the aggregation of misfolded α-synuclein and progressive spreading of the aggregates from a few discrete regions to wider brain regions. Although PD has been classically considered a movement disorder, a large body of clinical evidence has revealed the progressive occurrence of non-motor symptoms. Patients present visual symptoms in the initial stages of the disease, and accumulation of phospho-α-synuclein, dopaminergic neuronal loss, and retinal thinning has been observed in the retinas of PD patients. Based on such human data, we hypothesized that α-synuclein aggregation can initiate in the retina and spread to the brain through the visual pathway. Here, we demonstrate accumulation of α-synuclein in the retinas and brains of naive mice after intravitreal injection of α-synuclein preformed fibrils (PFFs). Histological analyses showed deposition of phospho-α-synuclein inclusions within the retina 2 months after injection, with increased oxidative stress leading to loss of retinal ganglion cells and dopaminergic dysfunction. In addition, we found accumulation of phospho-α-synuclein in cortical areas with accompanying neuroinflammation after 5 months. Collectively, our findings suggest that retinal synucleinopathy lesions initiated by intravitreal injection of α-synuclein PFFs spread to various brain regions through the visual pathway in mice.

**Supplementary Information:**

The online version contains supplementary material available at 10.1186/s40478-023-01575-0.

## Introduction

Parkinson’s disease (PD), the second-most common progressive neurodegenerative disorder [11], is classically recognized for its motor symptomatology, including rigidity, resting tremor and bradykinesia, which are attributable to dopaminergic deficiency caused by neuronal loss in the substantia nigra pars compacta (SNpc). The characteristic neuropathologic feature of PD is the presence of widespread intracellular inclusions of α-synuclein known as Lewy bodies [39]. This abnormal accumulation of fibrillar α-synuclein is thought to be crucial in the neurodegenerative process. Although these inclusions are found in the SNpc, they also extend to different areas, including many brain stem nuclei as well as the amygdala, forebrain, and neocortex [14]. Beyond motor clinical manifestations, PD patients also develop non-motor symptoms, including sleep disorders, depression, cognitive impairment and visual dysfunctions [47], some of which appear before movement impairments. Visual symptoms range from poor vision to reduced color discrimination, impaired contrast sensitivity, illusory visual symptoms, and abnormal saccadic eye movements [2]. Similar to the case for the SNpc, dopaminergic transmission is important in the retina; moreover, pathological retinal alterations, such as retinal thinning [21] and dopamine loss [36], have also been observed in PD patients. Notably, phospho-synuclein inclusions are not exclusively found in the brain but are also present in several layers of the retina [5, 35].

An examination of postmortem brains by Braak and colleagues led to advancement of the hypothesis that α-synuclein aggregates spread with disease progression [6]. Moreover, the presence of peripheral synucleinopathy lesions at early stages of the disease prompted a consideration of transsynaptic spreading of α-synuclein from sites outside the central nervous system (CNS), raising the question of whether PD may originate outside of the CNS [44]. After more than a decade of intense research, it is now widely accepted that, in the context of neurodegenerative diseases, pathogenic protein aggregates propagate within and beyond the CNS [24]. Spreading of α-synuclein aggregates in vivo has been modeled by injection of preformed fibrils (PFFs) into various tissues and organs [10]. Recent ophthalmological findings showing the presence of phospho-α-synuclein inclusions [4, 35], retinal degeneration [60], and a predisposition of the retina to protein misfolding [52] in PD patients have suggested that studying retinal pathology could offer greater insight into the origin and progression of PD [48, 60]. The study of retinal pathology could also support the potential use of the retina as a biomarker of this disease [23].

Motivated by human data showing retinal α-synuclein accumulation at early stages of PD and the distribution of synucleinopathy lesions in several brain regions of the visual pathway, we sought to test the hypothesis that aggregation of α-synuclein can be initiated in the retina and spread to the brain through the visual pathway. To this end, we studied the aggregation of α-synuclein in the mouse retina and spreading of its aggregates to the brain after intravitreal injection of α-synuclein PFFs. After intravitreal PFF injection, α-synuclein deposition within the retina was observed, together with phospho-α-synuclein aggregation and cellular, as well as dopaminergic deficits. In addition, we demonstrated that intravitreal injection of PFF triggers α-synuclein brain pathology in cortical areas accompanied by neuroinflammation.

## Materials and methods

### Generation of recombinant α-synuclein and preparation of mouse PFFs

For purification of mouse wild-type (WT) α-synuclein, *Escherichia coli* strain BL21 (DE3) was transformed with a pDdulGC vector encoding mouse WT α-synuclein and grown in LB medium at 37 °C. After the culture reached an optical density at 600 nm (OD_600_) of 0.6–0.7, protein expression was induced by adding isopropyl β-D-1-thiogalactopyranoside to a final concentration of 0.1 mM. After further incubation for 3 h at 37 °C, cells were harvested by centrifugation at 4000 × g for 15 min at 4 °C and sonicated. Following centrifugation of the lysate at 13,999 rpm (Supra R22, A50-8 rotor; Hanil Scientific, Gimpo, South Korea) for 20 min at 4 °C, the supernatant was boiled for 10 min, then centrifuged again under the same conditions. The resulting supernatant was filtered through 0.22-μm membranes and purified through anion exchange chromatography (HiTrap Q FF column; GE Healthcare Life Sciences, Chicago, IL, USA) by eluting with an NaCl gradient (concentration up to 1.0 M) in a base buffer of 20 mM Tris (pH 8.0). Eluted fractions containing α-synuclein, determined by monitoring using sodium dodecyl sulfate–polyacrylamide gel electrophoresis (SDS-PAGE), were then purified again by size-exclusion chromatography (Hiload 16/600 Superdex 200 pg column; GE Healthcare Life Sciences, Chicago, IL, USA). Pure fractions were pooled and dialyzed overnight against deionized water at 4 °C using a dialysis membrane with a 10-kDa cutoff and subsequently lyophilized.

Lyophilized α-synuclein was reconstituted in Dulbecco’s phosphate-buffered saline (DPBS; Gibco, a1285601, Carlsbad, CA, USA), filtered through a 100-kDa membrane (Nanosep, OD100C34, Pall Life Sciences, Port Washington, NY, USA), and incubated at 37 °C for 7 days with constant shaking at 1000 rpm. PFFs were stored at − 80 °C until the day of injection, at which time the fibrils were thawed at room temperature and sonicated for a total of 30 s (60 pulses, 1 s on, 1 s off) at 20% power (Vibracell VCX130; Sonics, Newtown, CT, USA).

### Transmission electron microscopy

For visualization of fibrils and characterization of the structure of the injected material, 5 mg/ml PFFs were diluted 1:50 in PBS, after which 10 µl was adsorbed onto 200-mesh carbon-coated copper grids (Electron Microscopy sciences, Hatfield, PA, USA), air dried for 5 min, and negative stained by incubating with 10 µl of 2% uranyl acetate (Cat. Number U1006, Spectrum Chemical, Brunswick, NJ, USA) for 5 min. Fibrils were observed using a JEM-1400 transmission electron microscope (JEOUL, Akishima, Tokyo Japan).

### Circular dichroism spectroscopy

Circular dichroism (CD) spectra of protein samples (monomers and fibrils) between 190 and 260 nm were obtained in 0.1-mm cells with a step resolution of 1.0 nm, bandwidth of 1.0 nm, and scan speed of 100 nm/min using a Chirascan Plus spectropolarimeter (Applied Photophysics, Leatherhead, Surrey, UK). All obtained spectra were averages of 10 separate measurements.

### Animals and intravitreal injection

All animal experiments were performed on male WT C57BL/6 J (000,664) mice in accordance with the standards of the Seoul National University Institutional Animal Care and Use Committee (IACUC) (SNU-200424–3-3). Eight-week-old male animals were anesthetized intraperitoneally with ketamine/xylazine, and an aperture was made through the sclera in both eyes with a 30-gauge needle. Thereafter, α-synuclein PFFs (5 µg) was intravitreally injected through the aperture using a 34-gauge Hamilton syringe (Hamilton, 207,434, Reno, NV, USA).

### Sample collection

At designated time points after injection, mice were deeply anesthetized by intraperitoneal (i.p.) administration of 200 mg/kg 1.25% Avertin (2,2,2 tribromoethanol, T48402; Sigma Aldrich, St. Louis, MO, USA) and perfused with an ice-cold 0.9% NaCl saline solution. Retinas, optic nerves, and brains were either dissected or fixed in 4% paraformaldehyde (PFA) for histological analysis.

### Retinal and optic nerve immunohistochemistry

After perfusion, eyes were enucleated, fixed in 4% PFA at 4 °C overnight and embedded in paraffin. Tissue Sections (4 µm thick) were deparaffinized with xylene and hydrated with a decreasing ethanol gradient (100%, 95%, 70%, 50%, dd-H_2_O), after which antigen recovery was performed by boiling in a water bath at 95 °C in citrate buffer (10 mM sodium citrate pH 6.0, 0.05% Tween-20 for 15 min. After allowing sections to cool for 20 min, they were washed twice with dd-H_2_O for 5 min each and treated with 3% H_2_O_2_ in Tris-buffered saline (TBS) for 30 min.

Sections were subsequently washed three times and blocked by incubating with 5% normal horse serum (Vector Laboratories, S-2000, Newark, CA, USA) in TBS containing 0.1% Triton X-100 (TBST) for 1 h at room temperature, followed by incubation with primary antibodies against α-synuclein (Syn1, 1:1000; BD Transduction, 610,787 clone 42/ α-synuclein, BD Biosciences, Franklin Lakes, NJ, USA), GFAP (1:5000; Abcam, ab7260, Cambridge, UK), Iba-1 (1:2000; Wako, 019–19,741, Osaka, Japan), pS129 synuclein (1:500; Abcam, EP1536Y clone, ab51253,), tyrosine hydroxylase (1:1000; Abcam, ab112) or 4-hydroxynonenal (1:1000, Abcam ab46545) and incubated in blocking solution overnight at room temperature in a humidified chamber. Afterwards, sections were washed with TBST and incubated with biotinylated secondary antibody (Vector Laboratories, 1:3000) in TBST for 1 h at room temperature, followed by detection using avidin–biotin-peroxidase complex (Vectastain ABC Kit; Vector Laboratories, PK6200) and staining with 3.3-diaminobenzidine (DAB) (ImmPACT DAB Substrate kit; Vector Laboratories).

To confirm phospho-antibodies specificity, prior to primary antibody incubation and after peroxidase quenching, sections were incubated with 4000 U/ml of lambda protein phosphatase (New England Biolabs, P0753S) for 30 min at 37 °C. Later samples were washed and continued with the standard staining procedure.

After DAB reaction, sections were counterstained with Nissl dye (Cresyl violet 0.1%), and images were captured using a Zeiss AX10 brightfield microscope (Carl Zeiss, Germany). Immunoreactivity (Intensity) or puncta were analyzed using Image J open-source software (NIH). For intensity measurements, all images were subjected to the same threshold. Mean intensity values were normalized to control for data graph representation.

### Retina immunofluorescence

Tissue Sections (4 µm thick) were deparaffinized with xylene and hydrated with a decreasing ethanol gradient (100%, 95%, 70%, 50%, dd-H_2_O), after which antigen recovery was performed by heating in a water bath at 95 °C in the citrate buffer (10 mM sodium citrate pH 6.0, 0.05% Tween-20) for 15 min. After allowing sections to cool for 20 min, sections were washed with TBST 3 times for 5 min each and blocked with 5% goat serum. Later, samples were incubated overnight at room temperature with primary antibodies against α-synuclein (Syn1, 1:1000; BD Transduction, 610,787 clone 42/ α-synuclein, BD Biosciences, Franklin Lakes, NJ, USA), pS129 synuclein (1:500; Abcam, EP1536Y clone, ab51253), AP2 α (Invitrogen, 3B5 MA1-872, Waltham, MA, USA) in 5% goat serum. After, sections were washed with TBST and incubated with anti-mouse/anti-rabbit secondary antibodies conjugated with Alexa 488 and Alexa 694, respectively, for 1 h at room temperature. Then, samples were washed and incubated with DAPI for 10 min and covered with Prolong Gold Antifade Mounting Media (Invitrogen, p36930, Waltham, MA, USA). Images were acquired with a Zeiss 700 confocal microscope equipped with a 40 × objective. All image processing was done using Zen Blue ver. 3.6.

### Whole-mount retina and quantification of retinal ganglion cell (RGC) density

Enucleated eyes were incubated in 4% PFA for 1 h and then retinas were detached by washing and dissecting in cold PBS. Four radial cuts towards the optic nerve entrance were made to flatten the retina for posterior mounting. Retinas were then blocked by incubating for 3 h with 5% goat serum in PBS containing 0.3% Triton X-100 at room temperature, then incubated with a primary antibody against the RNA-binding protein, RBPMS (Abcam, ab152101), at 4 °C overnight followed by incubation with secondary anti-rabbit Alexa 488 antibody in 0.3% Triton X-100 PBS for 4 h at room temperature. Tissue samples were then mounted on gelatin-coated slides and covered with Prolong Gold Antifade Mounting Media (Invitrogen, p36930, Waltham, MA, USA). Images were captured with a Zeiss 700 confocal microscope equipped with a 20 × objective. Four images per mice were obtained from the medial retina (500–600 µm from the optic nerve entry) for estimation of the density of RBPMS-positive cells. Cell quantification was performed blindly, and density was expressed as number of RBPMS-positive cells/ 1000 µm^2^. Visible blood vessel area was discarded from total working area used for quantification.

### Retina and brain lysate western blotting

Immediately after perfusion, eyes were enucleated and dissected to remove the retinas and optic nerves. Both retinas and optic nerves were transferred respectively to 150 µl and 100 µl of RIPA buffer with 1% protease (Sigma Aldrich, P8340, St Louis, MO, USA) and 1% phosphatase (Sigma Aldrich, P0044, St Louis, MO, USA) inhibitors. For brain western blots, brains were dissected into regions and then transferred to 1% Triton x-100 phosphate buffered saline (PBS) with 1% protease and 1% phosphatase inhibitors. Tissues were disrupted using a pestle homogenizer, and lysates were incubated on ice for one hour. After incubation, samples were centrifuged at 16.000 g for 15 min at 4 °C, and the soluble (supernatant) and insoluble (pellet) fractions were separated. Pellets were resuspended in the 1X Laemmli sample buffer and sonicated. Both the soluble and insoluble protein fractions were boiled in the Laemmli sample buffer for 10 min.

Protein samples were loaded and separated on 12% SDS–polyacrylamide gels and transferred to PVDF membranes. Membranes were blocked with 5% skim milk in PBST for 1 h and then incubated with primary antibodies: α-synuclein (1:1500, BD biosciences, #61,078), Caspase-3 (1:1000, Cell Signaling Technology, #9662, Danvers, MA, USA), β-actin (1:10,000, Sigma-Aldrich A5441, St Louis, MO, USA) in 1% BSA PBST overnight at 4 °C followed by incubation with the secondary antibody, goat anti-mouse or goat anti-rabbit (1:3000), for 1 h in 5% Skim Milk PBST. Blots were developed with ECL Solution (Amersham) and imaged using an Amersham Imager 600 (GE Healthcare). The levels of proteins were measured as optical density normalized by β-actin. Optical density was quantified using Multi Gauge v3.0 (Fujifilm, Japan).

### RNA extraction and qPCR

For RNA isolation from retinas, tissues from both eyes were homogenized in 1 ml of TRIzol reagent (Invitrogen, Waltham, MA, USA #15,596,026) using a RNAse free motorized tube pestle. After incubating 5 min at room temperature, tissue homogenates were treated with 200 µl of Chloroform and mixed vigorously by inversion. After 5 min at room temperature, samples were centrifuged at 12,000 rpm for 15 min at 4 °C. The aqueous phase was separated and mixed with an equal volume of 70% Ethanol and then transferred to a RNeasy column for RNA clean up following the manufacturer’s instructions, with an on-column DNase digestion (RNeasy Mini Kit, Qiagen, Hilden, NRW, Germany, #74,106). cDNA was synthetized by reversed transcription using iScript cDNA synthesis kit (Bio-Rad, Hercules, CA, USA, #1,708,891) following manufacturer's instructions. Proinflammatory target genes were amplified using iTaq Universal SYBR Green Supermix (Bio-Rad, #172–5121) on CFX Connect Real-Time PCR system (Bio-Rad, #1,855,201). Primer sequences are listed in Supplementary Table 1. Relative mRNA levels were calculated according to the 2-∆∆CT method. ∆Ct values were normalized to glyceraldehyde-3-phosphate dehydrogenase (GAPDH) values.

### Brain immunohistochemistry

After perfusion of mice with saline solution, brains were removed, fixed in 4% PFA for 48 h, and sectioned into 40-µm-thick slices. Free-floating sections were then washed with PBS and incubated with 3% H_2_O_2_ for 1 h. After peroxidase quenching, samples were washed and blocked with PBS containing 4% bovine serum albumin (BSA) in 0.1% Triton X-100 and incubated overnight at 4 °C with primary antibodies against phospho-synuclein pS129 (1:500; Abcam, ab51253), Iba-1 (1:500; Wako, 019–19,741), and TNF-α (1:250; Novus Biologicals, NBP1-19,532). The next day, sections were washed three times with PBST and then incubated first with biotinylated secondary antibodies (1:1000; Vector Laboratories) and then with avidin–biotin-peroxidase complex (Vectastain ABC kit; Vector Laboratories, PK6200), followed by staining with DAB (Sigma Aldrich, D5637).

After DAB reaction, brain sections were mounted on gelatin coated slides. Brain regions were identified using the Allen reference atlas, and images were captured using a Zeiss AX10 brightfield microscope (Carl Zeiss, Germany). The total area corresponding to the brain region of interest in the coronal slice was used for analysis. Immunoreactivity (Intensity), puncta and cell densities were analyzed using Image J open-source software (NIH). For intensity measurements, all images were subjected to the same threshold. For microglia density and TNF-α positive cell counts, images were subjected to background removal (mean filter and Rolling Ball algorithm) and same threshold and then counted with particle analysis function on Image J.

### Statistical analysis

Results in figures represent means ± s.e.m. Statistical significance was determined by calculating p-values with unpaired Student’s t-tests or ANOVA using GraphPad Prism 9.2.0 (GraphPad Software Inc., La Jolla, CA, USA). Graphs were drawn using GraphPad Prism 9.2.0.

## Results

### Uptake of α-synuclein PFFs into the retina after intravitreal injection

**Fig. 1:**
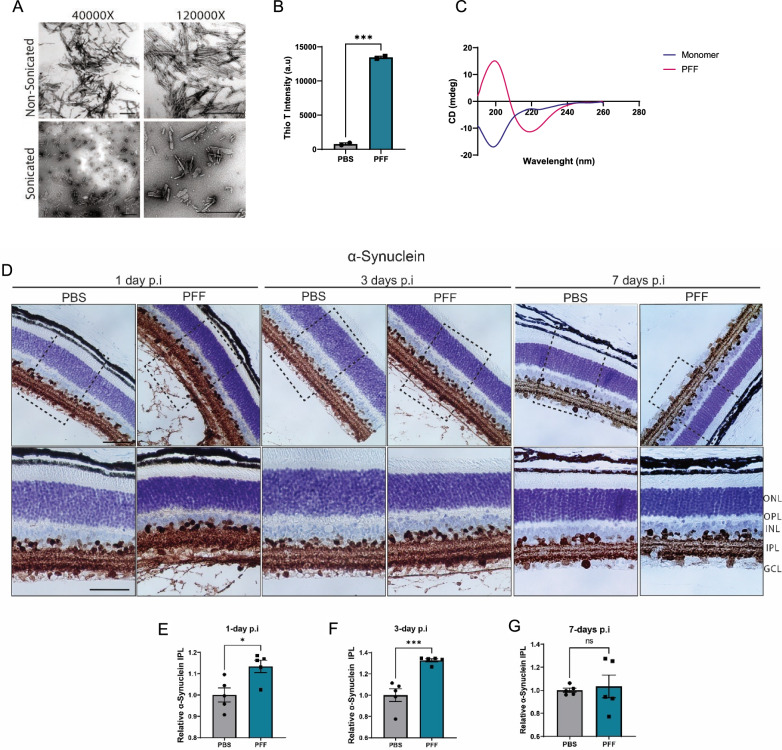
Uptake of α-synuclein fibrils following PFFs intravitreal injection. **A** TEM images of mouse PFFs. Scale bar: 500 nm. **B** Thioflavin-T intensity of fibrils at day 7 (n = 2 measurements). **C** CD spectra of α-synuclein monomers and PFFs. **D** Immunohistochemical staining of total α-synuclein in the retina of PFF-injected mice 1, 3, and 7 days after injection. **E**–**G** Quantification of immunoreactivity to α-synuclein in the IPL at 1 day (**E**), 3-days (**F**), and 7-days (**G**) after injection. Scale bar: 50 µm. Data in **E**, **F**, **G** are expressed as means ± s.e.m. (PBS, n = 5 mice; PFF, n = 5 mice; *p < 0.05, **p < 0.01, ***p < 0.001; Student’s t-test

To address whether pathological propagation of aggregates from the retina to the brain occurs in vivo, we injected mouse α-synuclein PFFs into the vitreous space of both eyes of C57BL/6J mice. As in previous reports [37, 40, 56], characterization of the generated fibrils using transmission electron microscopy (TEM), circular dichroism (CD) spectroscopy, and thioflavin T binding showed enriched β-sheet structures (Fig. 1A–C). Sonication-generated fragments were ~ 50–200 nm in size (Fig. 1A).

To confirm the uptake of α-synuclein into the retina after intravitreal injection, we performed an immunohistochemical analysis of retinal sections obtained 1-, 3- or 7-days post injection. PFF injection resulted in an increase in total α-synuclein in the inner plexiform layer (IPL) at 1 and 3 days after injection (Fig. 1D–F). Upon closer examination, we noted large amounts of α-synuclein in the vitreous space surrounding the lens and attached to the internal limiting membrane (ILM) in 1-day and 3-days sections (Fig. 1D), indicating that large amounts of fibrils were captured by the extracellular matrix components. However, the majority of the protein was degraded by day 7, at which point there were no significant differences in the levels of α-synuclein in the IPL (Fig. 1G) or residual α-synuclein in the vitreous space (Fig. 1D).

### Phosphorylated α-synuclein inclusions are found in the retinas of PFF-injected mice

**Fig. 2:**
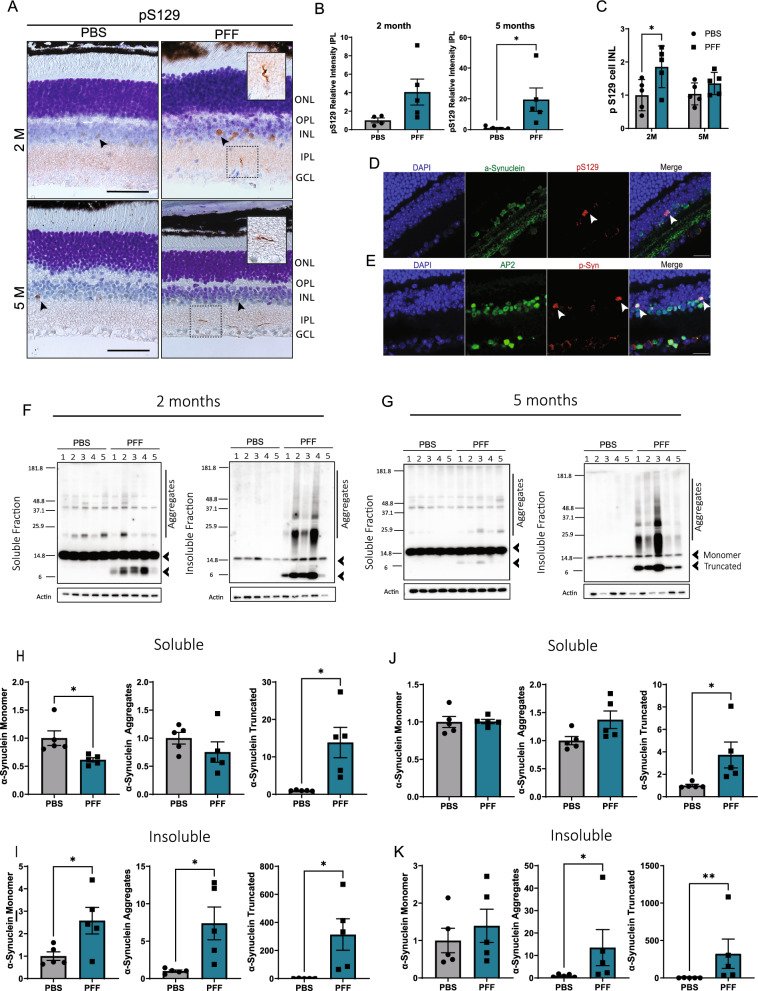
Intravitreal injection of α-synuclein fibrils leads to pS129 accumulation in the retina. **A** pS129-positive inclusions in the INL and IPL at 2 and 5 months after injection. Arrows indicate cell body inclusions in the INL. Close up shows neurite-like inclusions in the IPL. Scale bar: 50 µm. **B** Quantification of the relative immunoreactivity intensity of pS129 staining in the IPL at 2 months and 5 months post injection. **C** Quantification of pS129-positive cell inclusions in the border of the INL at 2 months and 5 months post injection. **D** Representative images of colocalization of α-synuclein and pS129 in the INL. **E** Colocalization of pS129 and amacrine cell marker AP2. Scale bar: 20 µm. **F**–**G** Representative western blot images of α-synuclein in the soluble and insoluble fractions obtained from the whole retinal lysates at 2 months (**F**) and 5 months (**G**) after injection. Arrowheads show quantified monomer and truncated α-synuclein. **H**–**K** Quantification of monomer, aggregated (above 20 kDa) and truncated α-synuclein in the soluble (**H**, **J**) and insoluble fractions (**I**, **K**) at 2 months (**H**, **I**) and 5 months (**J**, **K**) after injection. Data in **B**-**C** and **H**–**K** are expressed as means ± s.e.m. (PBS, n = 5 mice; PFF, n = 5 mice; *p < 0.05, **p < 0.01, ***p < 0.001; Student’s t-test and Two-way ANOVA)

The principal marker for α-synuclein aggregation in PD is phosphorylation at serine 129 (pS129) of this protein. To confirm that PFF injection generates pS129-α-synuclein inclusions in the retina, we performed immunohistochemical analyses. We observed pS129-positive structures in the IPL of the retina at 2 and 5 months after injection that resembled Lewy neurite-like inclusions (Fig. 2A-B). Localization of these inclusions was correlated with pS129-positive fibers detected in the retinas of PD patients [4]. In addition, we found pS129 structures in cell bodies, mainly localized to the border of the inner nuclear layer (INL) (Fig. 2A, arrows). Such inclusions were more visible at early time points after injection (Additional file 1: Supplementary Fig. 1A) and seemed to decrease with time. Although these structures were also present in control samples, their levels were significantly increased in 2-month PFF-injected retinas (Fig. 2C). To confirm that these pS129-positive inclusions corresponded to α-synuclein inclusions, we performed double staining for pS129 and total α-synuclein and found that both markers were co-localized (Fig. 2D). Moreover, such structures became undetectable following phosphatase treatment, further confirming their phosphorylated nature (Supplementary Fig. 1D). Given the localization of these pS129 cell body inclusions in the border of the INL, we questioned whether these were amacrine cells. To answer this question, we co-stained pS129 with the activating protein-2 (AP2), a marker for amacrine cells [58]. Co-localization of pS129 and AP2 confirmed the localization of pS129 to amacrine cells (Fig. 2E). Interestingly, these inclusions in the INL were more prominent at 2 months post injection (Fig. 2C) and became less visible at 5 months post injection (Fig. 2C). However, the amount of neurite-like pS129 inclusions in the IPL became more prominent after 5 months (Fig. 2B).

Western blot detection of α-synuclein also showed accumulation of aggregated α-synuclein in the insoluble fractions at 2 and 5 months (Fig. 2F-G). In addition, we observed the truncated α-synuclein (Fig. 2H-K) in both the soluble and insoluble fractions, likely derived from the injection material. Taken together, these results demonstrate that intravitreal injection of PFFs leads to an α-synuclein pathology in the retina.

### Retinal glial activation and proliferation are not prominent features following intravitreal injection of PFFs

**Fig. 3:**
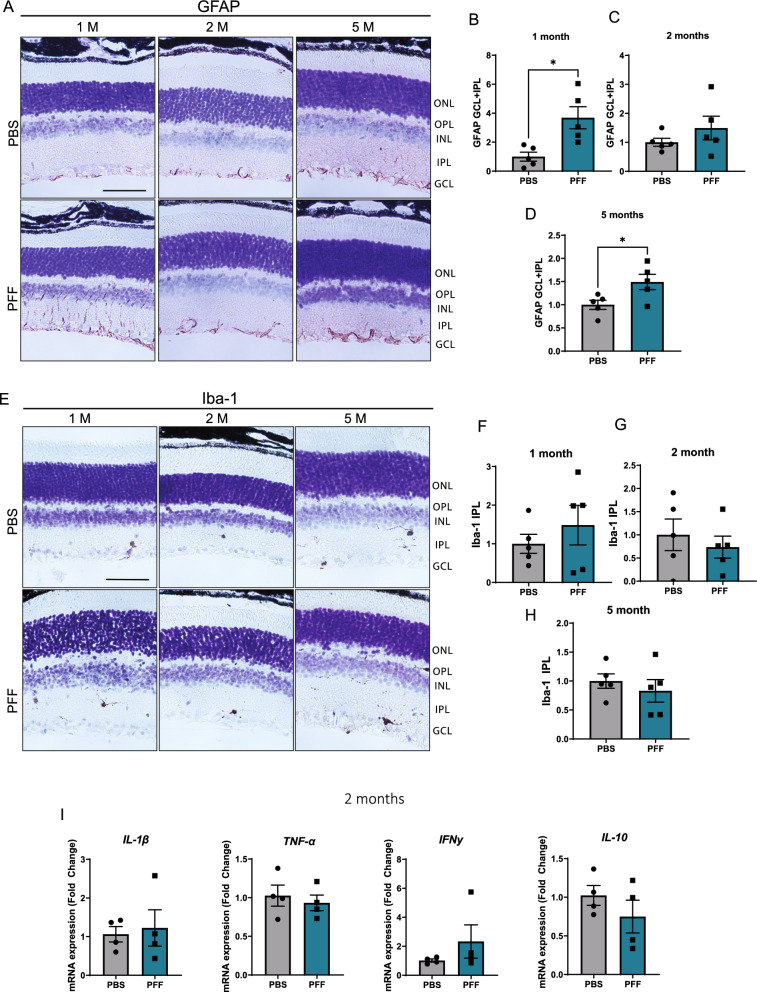
Glial responses and cytokine expression in the retina of PFF-injected mice. **A** Representative images of Müller glia identified based on GFAP immunoreactivity. **B**–**D** Quantification of GFAP intensity across the GCL and IPL layers of the retina at 1 month (**B**), 2 months (**C**), and 5 months (**D**) after injection of PFFs. **E** Representative images of Iba-1–immunostained microglia. **F**–**H** Quantification of Iba-1 intensity in the IPL layers at 1 month (**F**), 2 months (**G**), and 5 months (**H**). **I** mRNA expression of proinflammatory cytokines Il-1β, TNF- α, IFNγ and IL-10 in the retina of 2 months after injection, quantified by RT-qPCR. Data values are expressed as means ± s.e.m. (Data from (**A**–**D**): PBS, n = 5 mice; PFF, n = 5 mice; **I**: PBS, n = 4 mice; PFF, n = 4 mice *p < 0.05, **p < 0.01, ***p < 0.001; Student’s t-test)

α-Synuclein accumulation is usually accompanied by activation and proliferation of glial cells [27]. To analyze activation of Müller glia and microglia, we assessed the expression of glial fibrillary acid protein (GFAP) and ionized calcium-binding adapter molecule 1 (Iba-1), respectively, in retinal sections. Both PBS- and PFF-injected mice showed glial activation, with glial processes extending from the ganglion cell layer (GCL) through the IPL; however, no differences between the experimental groups were seen at either 1 or 3 days after injection (Supplementary Fig. 2). Later, compared with PBS control samples, PFF-injected retinas showed an increase in Müller glia activation 1 month after injection, a response that became not significant at 2 months (Fig. 3A-C). In addition, GFAP immunoreactivity was greater at 5 months post injection, although unlike the initial 1-month response, glial activation was mostly restricted to the GCL at this later time point (Fig. 3D). On the other hand, Iba-1 immunoreactivity revealed very few microglia in retinas from either PBS- or PFF-injected animals, and we observed no differences in morphology or number at any time point examined (Fig. 3E-H). To confirm the absence of inflammatory responses, we analyzed the levels of proinflammatory cytokines in the retina 2 months after injection. We found no changes in mRNA expression levels of IL-1β, TNF-α, IFNγ and IL-10 (Fig. 3I). Overall, these results suggest that glial activation in the retina is not a major feature after PFF injection.

### Neuronal and dopaminergic deficits in the retinas of PFF-injected mice

**Fig. 4:**
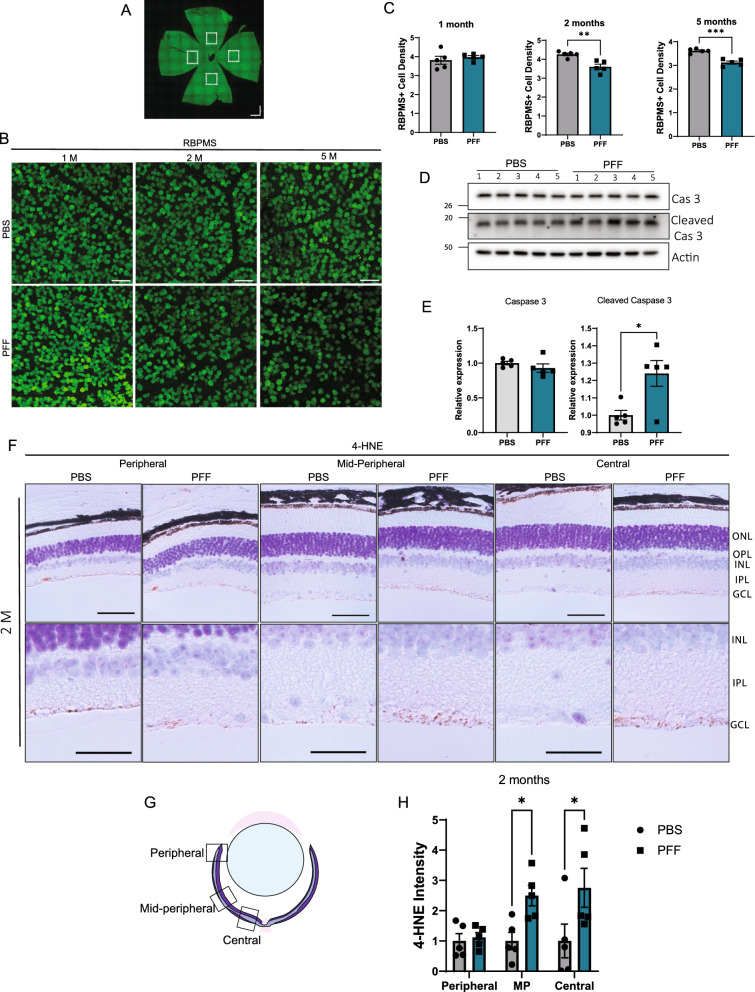
Intravitreal injection of PFFs leads to oxidative stress and RGC loss. **A** Representative image of the whole mount retina. Images were taken from 500–600 µm from the optic nerve. **B** Representative images of RBPMS immunostaining in the retinal whole mounts. Scale bar: 50 µm. **C** Quantification of RBPMS-positive cell numbers at 1, 2, and 5 months post injection. Cell density is expressed as cells per 1000 µm.^2^. **D** Representative western blot images of the retinal lysates for the total and cleaved caspase 3 at 2 months post injection. **E** Quantification of the total and cleaved caspase 3 in the retina of injected mice. **F** Representative images of 4-HNE immunoreactivity in the retinal sections at 2 months after injection. Positive staining is observed mainly in the GCL. Scale bar: 50 µm. **G** Diagram of the peripheral, mid-peripheral and central retina regions. **H** Quantification of 4-HNE intensity in the GCL layer in the peripheral, midperipheral and central retina. Data are expressed as means ± s.e.m, relative to control (PBS, n = 5 mice, PFF n = 5 mice; *p < 0.05, **p < 0.01, ***p < 0.001; Student’s t-test and Two-way ANOVA)

Next, we evaluated whether PFF injection led to neuronal degeneration in the retina. First, we quantified retinal ganglion cell (RGC) density in retinal whole mounts using the marker, RBPMS (RNA-binding protein, mRNA-processing factor). RGCs are a known target of degeneration in many neurodegenerative diseases, including PD [20]. An examination of whole mounts showed that, after 2 and 5 months, the number of RBPMS-positive cells decreased in the midperipheral retinas (Fig. 4A) from PFF-injected mice compared with those from control mice (Fig. 4B–C). To confirm the cells death, we performed western blot analysis for caspase 3 and observed an increase in the expression of the cleaved form of caspase 3 (Fig. 4E). Cellular oxidative stress has been associated with α-synuclein accumulation and cell toxicity [41]. To further understand how α-synuclein was triggering cell death, we stained the retinal sections against lipid peroxidation marker, 4-hydroxynonenal (4-HNE). Immunohistochemical staining revealed an increase in 4-HNE at 2 months, mostly localized in the GCL in the midperipheral and central retinas (Fig. 4F-H), which correlates with the location of RBPMS cell loss. The rise of oxidative stress could be observed from 1-month post injection samples in the midperipheral and central retinas (Supplementary Fig. 3A, C) and decreased by 5 months (Supplementary Fig. 3B, D).

**Fig. 5:**
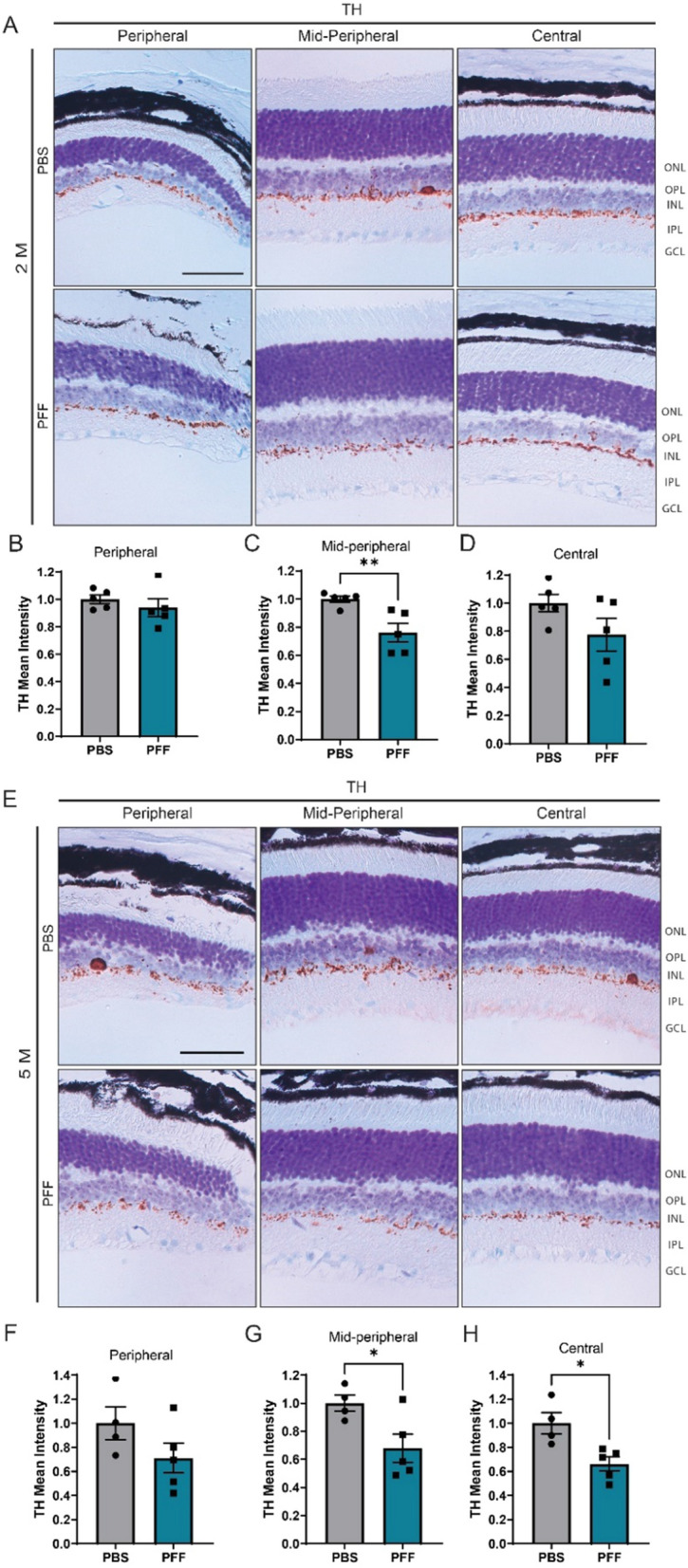
Intravitreal injection of PFFs leads to a decrease in TH levels in the central and midperipheral retina. **A**, **D** Representative images of TH immunostaining in the retina at 2 months (**A**) and 5 months (**E**) after injection. **B**–**D**, **F**–**H** Quantification of the intensity of TH immunoreactivity in the border between the INL and IPL from the peripheral, midperipheral and central retina at 2 (**B**-**D**) and 5 months (**F**–**H**) post injection. Scale bar: 50 µm. Data are expressed as means ± s.e.m, relative to control (PBS, n = 5 mice, PFF n = 5 mice; *p < 0.05, **p < 0.01, ***p < 0.001; Student’s t-test)

Dopaminergic cell loss has also been described in the retina of PD patients [36]. To test for the presence of such changes in the retinas of PFF-injected mice, we immunostained retinal sections for tyrosine hydroxylase (TH), which catalyzes the rate-limiting step in the synthesis of dopamine. PFF-injected mice exhibited a reduction in TH levels in the IPL layer at both 2 and 5 months (Fig. 5A-H). At 2 months, the decrease in TH was mostly observed in the midperipheral retina (Fig. 5C). However, reaching 5 months, differences were also observed closer to the optic nerve, in the central retina (Fig. 5H). Some of these alterations are related with changes described in patients that exhibited dopaminergic loss and inner retinal layer thinning in the parafoveal retina [22].Taken together, these results show that PFF injection leads to cellular and dopaminergic deficits in the retina.

### Accumulation of α-synuclein in the retina leads to microglia activation in the optic nerve

**Fig. 6:**
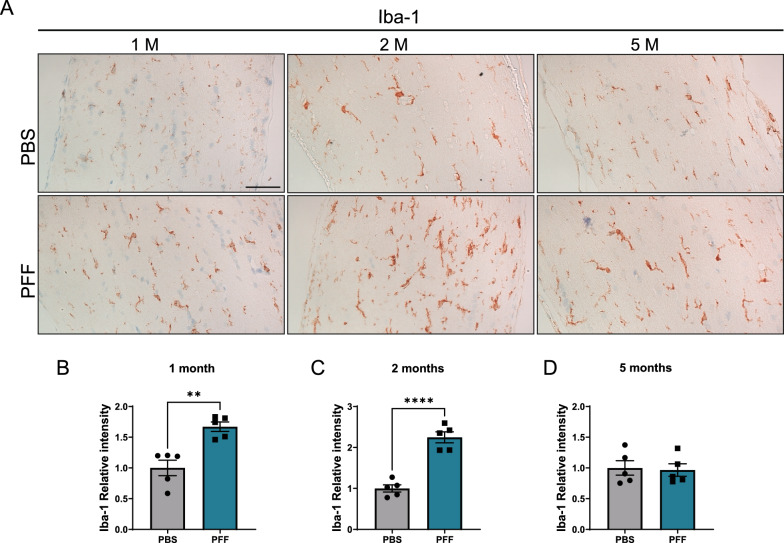
Microgliosis in the optic nerve of PFF-injected mice. **A** Representative images of Iba-1 immunoreactivity in the optic nerve 1, 2, and 5 months after injection. **B**–**D** Quantification of Iba-1 immunoreactivity in the optic nerve. Scale bar: 50 µm. Data are expressed as means ± s.e.m, relative to control (PBS, n = 5; PFF, n = 5; *p < 0.05, **p < 0.01, ***p < 0.001, ****p < 0.000; Student’s t-test)

Eyes are anatomically connected to the brain through the optic nerve, an extension of RGC axons that relays visual information from the retina to the brain. Optic nerve fibers are vulnerable to injury, and optic neuritis is known to trigger RGCs loss. Moreover, optic nerve pathology has also been described in PD [38, 46]. To better understand changes in the optic nerve after PFF injection, we performed an immunohistochemical analysis of Iba-1 in microglial cells (Fig. 6A) and found an increase in Iba-1 in optic nerves at 1 and 2 months (Fig. 6B, C). However, consistent with the increase in oxidative stress (Fig. 4F, Supplementary Fig. 3B), the microglial activation was not detected at 5 months (Fig. 6D), demonstrating that optic inflammation is transient.

### Cortical pathology in the brains of PFF-injected mice

**Fig. 7:**
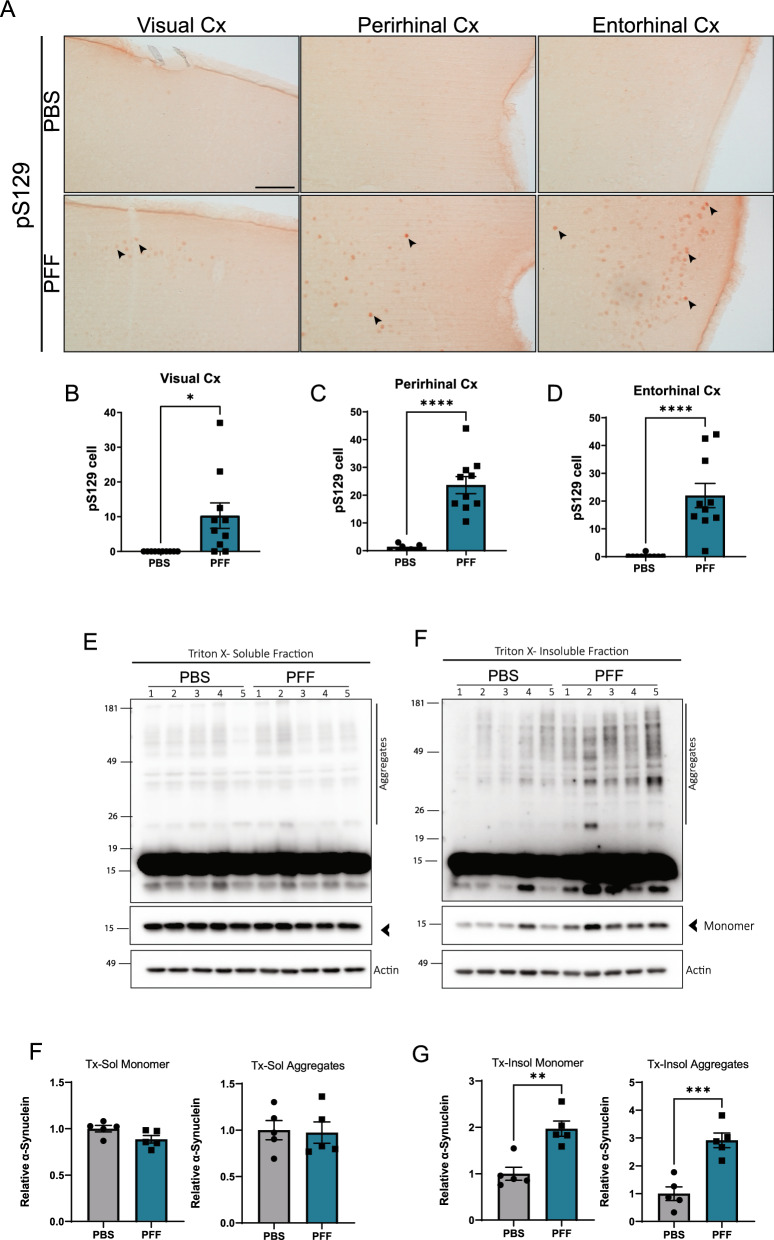
Intravitreally injected α-synuclein causes brain α-synuclein accumulation 5 months after injection**. A** Representative images of pS129 immunostaining in the visual, perirhinal, and entorhinal cortices at 5 months after injection. **B**–**D** Quantification of pS129-positive cell puncta in the cortical areas. Numbers represent the total number of puncta in the indicated region from one coronal brain slice. Visual Cortex (**B**), Perirhinal Cortex (**C**) and Entorhinal Cortex (**D**). Brain areas were delimited using the Allen Atlas as reference. Scale bar: 100 µm. Data are expressed as means ± s.e.m. (PBS, n = 10; PFF, n = 10; *p < 0.05, **p < 0.01, ***p < 0.001; Student’s t-test). **E**–**F** Representative western blots of α-synuclein in the rhinal cortex at 5 months post injection. **F**, **G** Quantification of α-synuclein monomer and aggregates (above 20 kDa) in the triton-x soluble **E** and insoluble **F** fractions. Data are expressed as means ± s.e.m, relative to control (PBS, n = 5; PFF, n = 5; *p < 0.05, **p < 0.01, ***p < 0.001; Student’s t-test)

Finally, we asked whether intravitreal injection of PFFs could cause propagation of α-synuclein to the brain. To this end, we examined the presence of pS129 in several brain areas associated with the visual pathway. First, we examined the superior colliculus (SC) and lateral geniculate nucleus (LGN). We did not observe any pS129-positive structures at any of these regions (Supplementary Fig. 4). However, we found pS129-positive inclusions in the visual cortex and associated perirhinal and entorhinal cortices of intravitreal PFF-injected mice (Fig. 7A, B). To confirm these results, we analyzed the Triton x-soluble and insoluble fractions from the rhinal cortex (including the entorhinal and perirhinal areas) and found an increase of both the insoluble monomer and high molecular weight α-synuclein, demonstrating that intravitreal injection of PFF causes aggregation of α-synuclein in the brain areas.

**Fig. 8:**
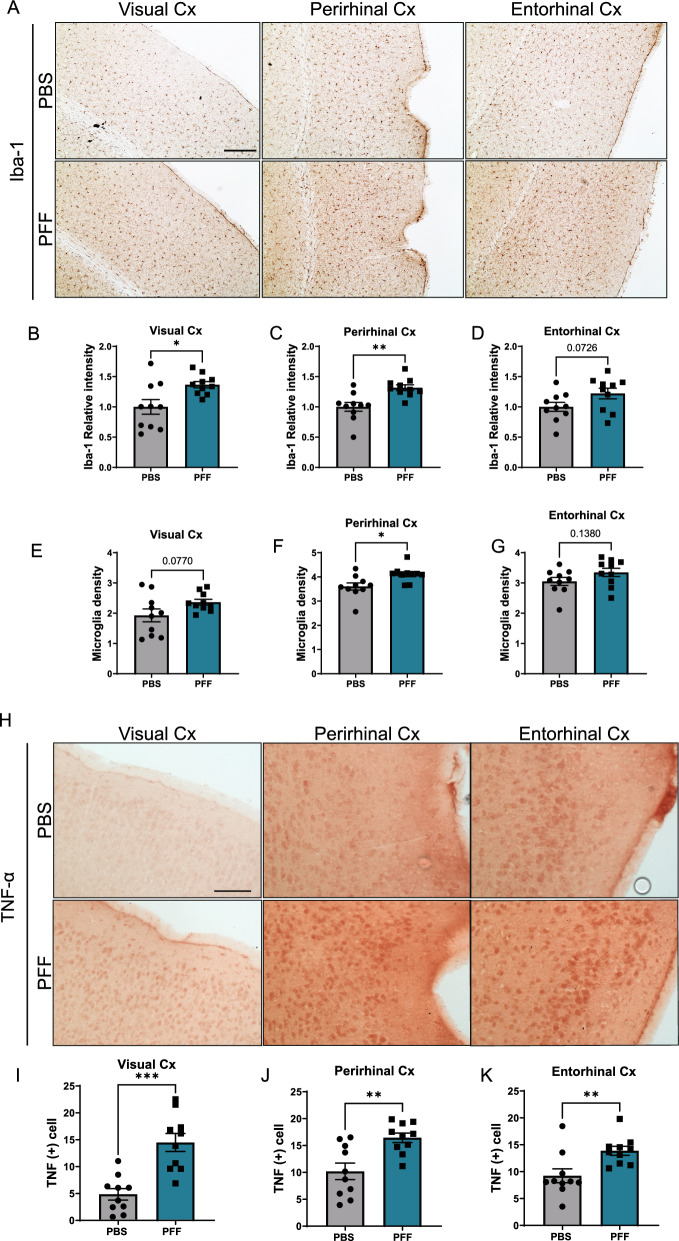
Intravitreally injected α-synuclein causes the cortical inflammation 5 months after injection. **A** Representative images of microglia (Iba-1–positive cells) in the visual cortex, perirhinal cortex, and entorhinal cortex. **B**–**D** Quantification of the optical density of Iba-1 immunostaining in the cortical areas. **E**–**G** Microglial density in the visual, perirhinal and entorhinal cortices, normalized per area and expressed as microglia cell number per 10,000 µm^2^. (H) Representative images of TNF-α immunostaining in the cortical areas. (I-K) Quantification of TNF-α–positive cell counts in the cortical areas per 10,000 µm^2^. Scale bar: 100 µm. Data are expressed as means ± s.e.m. (PBS, n = 10; PFF, n = 10; *p < 0.05, **p < 0.01, ***p < 0.001; Student’s t-test)

We then assessed if inflammatory changes occurred in these regions (Fig. 8A-C). Quantification of microglial densities in these areas showed that the number of microglia was greater in the perirhinal cortex of PFF-injected animals (Fig. 8C). Microglial density in the visual cortex of PFF-injected mice also trended higher, although this difference did not reach statistical significance. In addition, immunoreactivity for TNF-α was increased in the cortices of PFF-injected animals (Fig. 8H-I), suggesting that intravitreal injection of PFF leads to brain inflammation.

## Discussion

In this work, we injected α-synuclein PFFs into the vitreous space and examined the pathological effects of protein aggregation in the retina and subsequent spreading of aggregates to the brain. Our results showed that the mouse retina displays phospho-α-synuclein inclusions that closely resemble Lewy neurites. Previous reports [54] indicated that α-synuclein propagation from the eye was not possible owing to the lack of internalization of fibrils into the inner retina, resulting in the absence of intraretinal phospho-α-synuclein inclusions. Consistent with these observations, we detected large amounts of injected fibrils attached to the ILM, but in contrast to this previous study [54], we were also able to detect an increase in total α-synuclein within the IPL, suggesting actual uptake into the retina. A possible explanation for the differences between our study and this previous study is that we used fibrils derived from the mouse form of α-synuclein, which have been shown to have greater seeding properties in vivo than human α-synuclein PFFs [28]. We also did not tag or attach any probes to the protein that could modify protein absorption or aggregation properties [49, 57].

Unlike the Lewy neurite-like structures in the IPL layer, which were found only in PFF-injected animals, phospho-α-synuclein cell body inclusions were observed in the border of the INL layer in both PBS- and PFF-injected animals. Similar inclusions have been observed in retinas of α-synuclein transgenic mice [29]. Furthermore, histological examinations of retinas from aged individuals have shown similarly localized ubiquitinated synuclein deposits in the bodies of cells [25]. However, a previous examination of phosphorylated α-synuclein in PD patients showed no evidence for phospho-α-synuclein deposition in control subjects [35]. The reason for the occurrence of inclusions in the INL layer is not yet clear. However, it does not seem that these inclusions are responsible for the spread of α-synuclein aggregates into the brain because PBS-injected animals showed no α-synuclein pathology in the brain, despite the presence of α-synuclein inclusions in the INL layer.

The appearance of phospho-α-synuclein in the brain after PFF injection is usually accompanied by increased proliferation and activation of inflammatory cells, such as microglia and astrocytes [12]. Our results showed mild activation of Müller glia with no signs of microglia proliferation or induction of proinflammatory cytokine release, indicating that inflammation is not a primary characteristic of the retina in this model. The lack of retinal inflammatory responses was also reported in an α-synuclein transgenic model, in which no difference was detected in Müller glia or microglia compared with non-transgenic controls, despite widespread α-synuclein protein accumulation and synaptic degeneration in the inner retina [17, 53]. These findings suggest that inflammation is not a main feature of retinal pathology in synucleinopathy models.

Our data also revealed that injection of PFF resulted in the loss of RGCs. These findings are in accord with the results of α-synuclein overexpression in the retina after AAV injection, which also showed a decrease in RGCs 2 months after injection [30]. We observed that RGCs loss could be associated with the increase in oxidative stress caused by α-synuclein in the RGC layer, leading to apoptotic cell death. These results show that α-synuclein pathology mainly affects this neuronal population in the retina.

We also observed a decrease in the levels of TH in our model, confirming the role of retinal α-synuclein aggregation in dopaminergic degeneration and providing clues to visual dysfunction in PD. In addition, we noticed that the midperipheral and central retinas had increased susceptibility to dopaminergic alterations compared with the peripheral retina. The previous studies have reported dopaminergic loss and synaptic alterations in the retinas of PD patients [1, 36, 62] and described a correlation between nigral dopaminergic loss and parafoveal inner retinal layer thinning [22]. Nevertheless, a more detailed characterization of the dopaminergic and morphological alteration in AII amacrine cells caused by α-synuclein aggregation is needed to better understand regional susceptibility in the retina.

In the current study, we report the spreading of phosphorylated α-synuclein pathology to the brain after intravitreal injection of PFFs in naïve mice, suggesting the potential for retinal initiation of PD pathology. Although our study is the first to demonstrate the propagation of α-synuclein pathology from the retina to the brain, a previous study by Mammadova et al*.* [29] also suggested a relationship between the brain and the retina in the spread of synucleinopathy. This latter study showed acceleration of α-synuclein accumulation in eyes after intracerebral inoculation of a brain homogenate prepared from α-synuclein transgenic mice.

Consistent with the theory that spreading of protein aggregates proceeds through anatomical neural connections [32, 45], we found α-synuclein inclusions in cortical areas associated with the visual pathway, demonstrating that intravitreally injected PFFs can trigger retina-to-brain propagation of α-synuclein pathology. However, we found no trace of phospho-α-synuclein in some brain areas that are also connected to the optic nerve, including the lateral geniculate nucleus and superior colliculus. The underlying mechanism for this regional selectivity is not fully understood. Modeling of the dynamics of α-synuclein spreading have indicated that, in addition to connectivity, expression patterns of α-synuclein are a major contributor to pathogenic protein spreading [15, 42]. Consistent with this model, cortical regions that show α-synuclein pathology in our study have higher endogenous levels of α-synuclein than the regions that do not [26].

According to Braak’s hypothesis, Lewy body pathology starts in the lower brainstem and olfactory bulb and spreads progressively to cortical structures [6]. However, clinical studies have suggested that a significant proportion of cases do not follow this pattern [55]. Such heterogeneity among PD patients could be explained by the existence of a variety of starting points for the spread of the pathology. These multiple initiation sites could be in play even in a single patient, generating complex clinical and pathological outcomes. The current study suggests that the retina is one such site that can initiate α-synuclein aggregation and spread the pathology to the brain, accounting for some of the cortical pathology. Clinical evidence indicates that the onset of visual symptoms precedes initiation of motor symptoms in PD [47]. Moreover, thinning of the inner retina [1, 21, 51] and retinal microvascular impairment [19, 43] have been observed in early PD patients. These clinical data support our hypothesis that the eye may be an initiation point for PD pathology.

The retina is exposed to various environmental stresses and deleterious insults [13] and therefore is susceptible to abnormal protein misfolding and aggregation [50]. Several degenerative eye conditions, including retinitis pigmentosa, cataracts [34], age-related macular degeneration and glaucoma, are known to be associated with proteasome inhibition [31] and autophagy defects [33, 59], which lead to protein accumulation [52]. Recently, two population-based retrospective cohort studies found an increased risk of PD among patients with age-related macular degeneration [8, 9], further suggesting a relationship between eye diseases and the development of PD.

It is important to mention the microgliosis observed in the optic nerve after PFF injections in our model animals, as optic neuropathy is associated with diseases such as Alzheimer’s disease and PD [7]. It is known that optic nerve injury leads to an increase in NLRP3 activation and secondary degeneration of the visual cortex [61]. Interestingly, recent studies have shown that inflammation plays a major role in the propagation of protein aggregates [3, 16, 18]. Taken together, the available evidence suggests the possibility that α-synuclein pathology in the brains of mice intravitreally injected with PFFs may be mediated by optic nerve inflammation.

## Conclusions

**Fig. 9:**
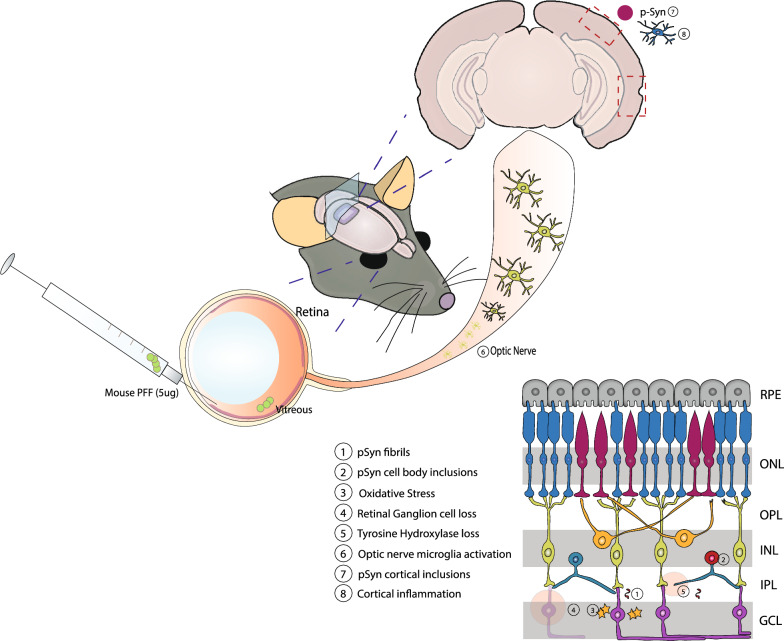
α-synuclein propagation from the retina to cortical brain areas after intravitreal injection of PFFs. Injection of α-synuclein PFFs into the vitreous space caused deposition of pS129-positive neuritic (1) and cell body (2) inclusions in the IPL and INL, respectively. Accumulation of α-synuclein led to oxidative stress (3) and triggered apoptotic cell death of the RGCs (4), loss of tyrosine hydroxylase (5), and activation of optic nerve microglia (6). Injection of PFFs also resulted in aggregation of α-synuclein in the cortex (7) accompanied by inflammatory responses (8)

Intravitreal injection of α-synuclein PFFs causes accumulation of phospho-α-synuclein in the IPL layer of the retina, leading to oxidative stress, RGC loss and dopaminergic deficits. This model recapitulates the main aspects of retinal pathology in PD patients. Additionally, injection of PFFs results in inflammation in the optic nerve and cortical areas. Finally, phospho-α-synuclein–positive inclusions are present in visual and rhinal cortices 5 months after injection, demonstrating retina-to-brain spreading of α-synuclein pathology (Fig. 9). Thus, our study provides evidence for retina-initiated spreading of synucleinopathy to the brain and establishes a new animal model for studying synucleinopathy spreading.

### Supplementary Information

**Additional file 1.** Supplementary Table 1 (Primer Sequences) and Supplementary Figures 1–4.

## Data Availability

The datasets used and/or analyzed during the current study are available from the corresponding author on reasonable request.

## Abbreviations

PD: Parkinson’s disease
CNS: Central nervous system
SNpc: Substantia nigra pars compacta
WT: Wild type
PFFs: Preformed fibrils
TEM: Transmission electron microscopy
CD: Circular dichroism
IPL: Inner plexiform layer
ILM: Internal limiting membrane
pS129: Phosphoserine 129
INL: Inner nuclear layer
AP2: Activating protein-2
GFAP: Glial fibrillary acidic protein
Iba-1: Ionized calcium-binding adapter molecule 1
RBPMS: RNA binding protein with multiple spacing
TH: Tyrosine hydroxylase
RGCs: Retinal ganglion cells
GCL: Ganglion cell layer
TNF-α: Tumor necrosis factor alpha
SC: Superior colliculus
LGN: Lateral geniculate nucleus
