# Correction: Retina-to-brain spreading of α-synuclein after intravitreal injection of preformed fibrils

**DOI:** 10.1186/s40478-024-01816-w

**Published:** 2024-07-02

**Authors:** Dayana Pérez-Acuña, Ka Hyun Rhee, Soo Jean Shin, Jeeyun Ahn, Jee-Young Lee, Seung-Jae Lee

**Affiliations:** 1 Department of Biomedical Sciences, Seoul National University College of Medicine, 103 Daehak-Ro, Jongro-Gu, Seoul, 03080 Korea; 2 Department of Ophthalmology, College of Medicine, Seoul Metropolitan Government-Seoul National University Boramae Medical Center, Seoul National University, Seoul, South Korea; 3 Department of Neurology, Seoul Metropolitan Government-Seoul National University Boramae Medical Center, Seoul National University College of Medicine, Seoul, South Korea; 4 Neuroscience Research Institute, Seoul National University College of Medicine, Seoul, South Korea; 5 Convergence Research Center for Dementia, Seoul National University College of Medicine, Seoul, South Korea; 6 Neuramedy, Seoul, South Korea; 7 Present Address: Department of Biochemistry, Molecular Biology and Biophysics, University of Minnesota, Minneapolis, MN 55455 USA

**Correction: Acta Neuropathologica Communications (2023) 11:83** 10.1186/s40478-023-01575-0

Following publication of the original article [1], the authors have identified that an error was present in two of the images. Figure 1D and Figure 5E have incorrect representative images. The authors have revised the data and verified that such an error does not affect the integrity and accuracy of the content presented in the article.

For figure 1D 7 days p.i PBS and PFF, although images belong to the indicated experimental group, lower magnification images do not correspond to the zoomed magnification images. For clarification, correct images in the upper panel for 7 days p.i are now included.

In the case of Figure 5, Figure 5A and E for 2 Months and 5 Months PBS Central contain a duplicated image. In this correction, the image in Fig. 5E panel has been corrected with the accurate representative image for 5 M PBS Central.

The incorrect Fig. 1(D):

**Fig. 1:**
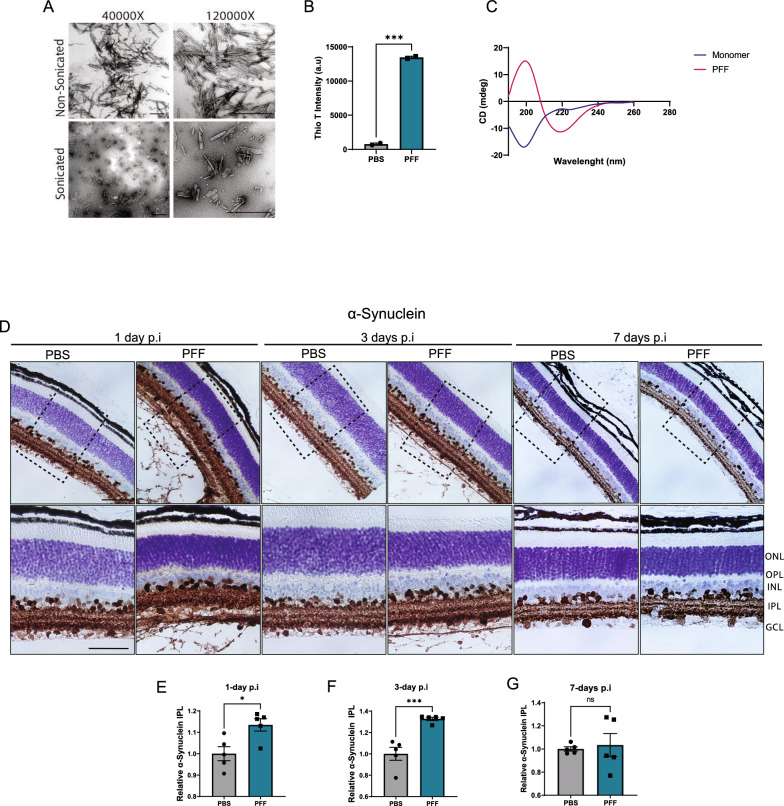
Uptake of α-synuclein fibrils following PFFs intravitreal injection. **A** TEM images of mouse PFFs. Scale bar: 500 nm. **B** Thioflavin-T intensity of fibrils at day 7 (n = 2 measurements). **C** CD spectra of α-synuclein monomers and PFFs. **D** Immunohistochemical staining of total α-synuclein in the retina of PFF-injected mice 1, 3, and 7 days after injection. **E–G** Quantification of immunoreactivity to α-synuclein in the IPL at 1 day (**E**), 3-days (**F**), and 7-days (**G**) after injection. Scale bar: 50 μm. Data in **E**, **F**, **G** are expressed as means ± s.e.m. (PBS, n = 5 mice; PFF, n = 5 mice; *p < 0.05, **p < 0.01, ***p < 0.001; Student’s t-test

The correct version of Figure 1(D):

**Fig. 1:**
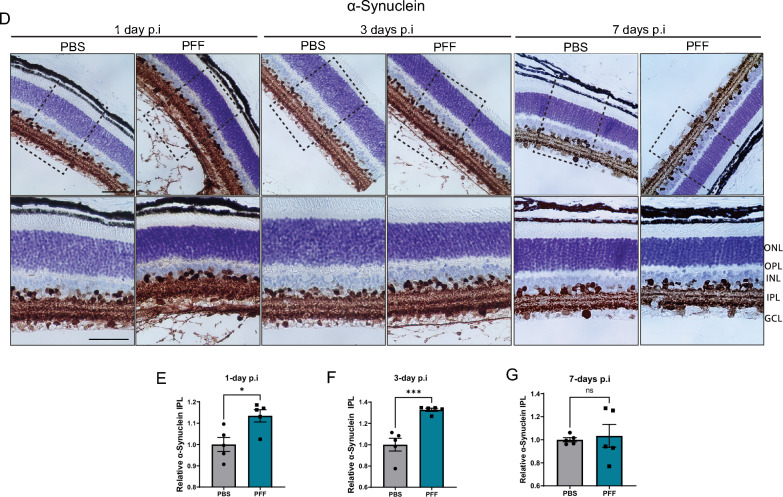
Uptake of α-synuclein fibrils following PFFs intravitreal injection. **A** TEM images of mouse PFFs. Scale bar: 500 nm. **B** Thioflavin-T intensity of fibrils at day 7 (n = 2 measurements). **C** CD spectra of α-synuclein monomers and PFFs. **D** Immunohistochemical staining of total α-synuclein in the retina of PFF-injected mice 1, 3, and 7 days after injection. **E**–**G** Quantification of immunoreactivity to α-synuclein in the IPL at 1 day (**E**), 3-days (**F**), and 7-days (**G**) after injection. Scale bar: 50 μm. Data in **E**, **F**, **G** are expressed as means ± s.e.m. (PBS, n = 5 mice; PFF, n = 5 mice; *p < 0.05, **p < 0.01, ***p < 0.001; Student’s t-test

The incorrect Figure 5(E):

**Fig. 5:**
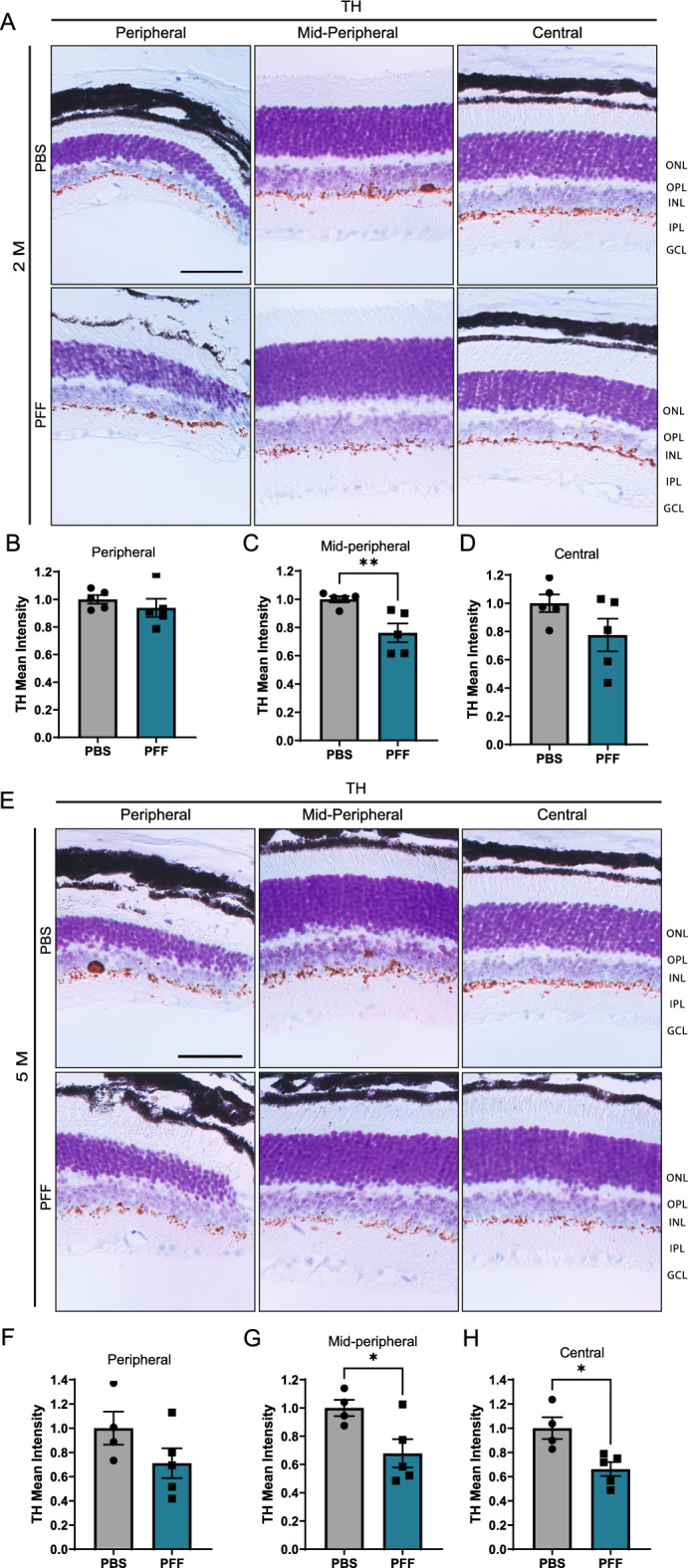
Intravitreal injection of PFFs leads to a decrease in TH levels in the central and midperipheral retina. **A**, **D** Representative images of TH immunostaining in the retina at 2 months (**A**) and 5 months (**E**) after injection. **B–D**, **F–H** Quantification of the intensity of TH immunoreactivity in the border between the INL and IPL from the peripheral, midperipheral and central retina at 2 (**B–D**) and 5 months (**F–H**) post injection. Scale bar: 50 μm. Data are expressed as means ± s.e.m, relative to control (PBS, n = 5 mice, PFF n = 5 mice; *p < 0.05, **p < 0.01, ***p < 0.001; Student’s t-test)

The correct version of Figure 5(E):

**Fig. 5:**
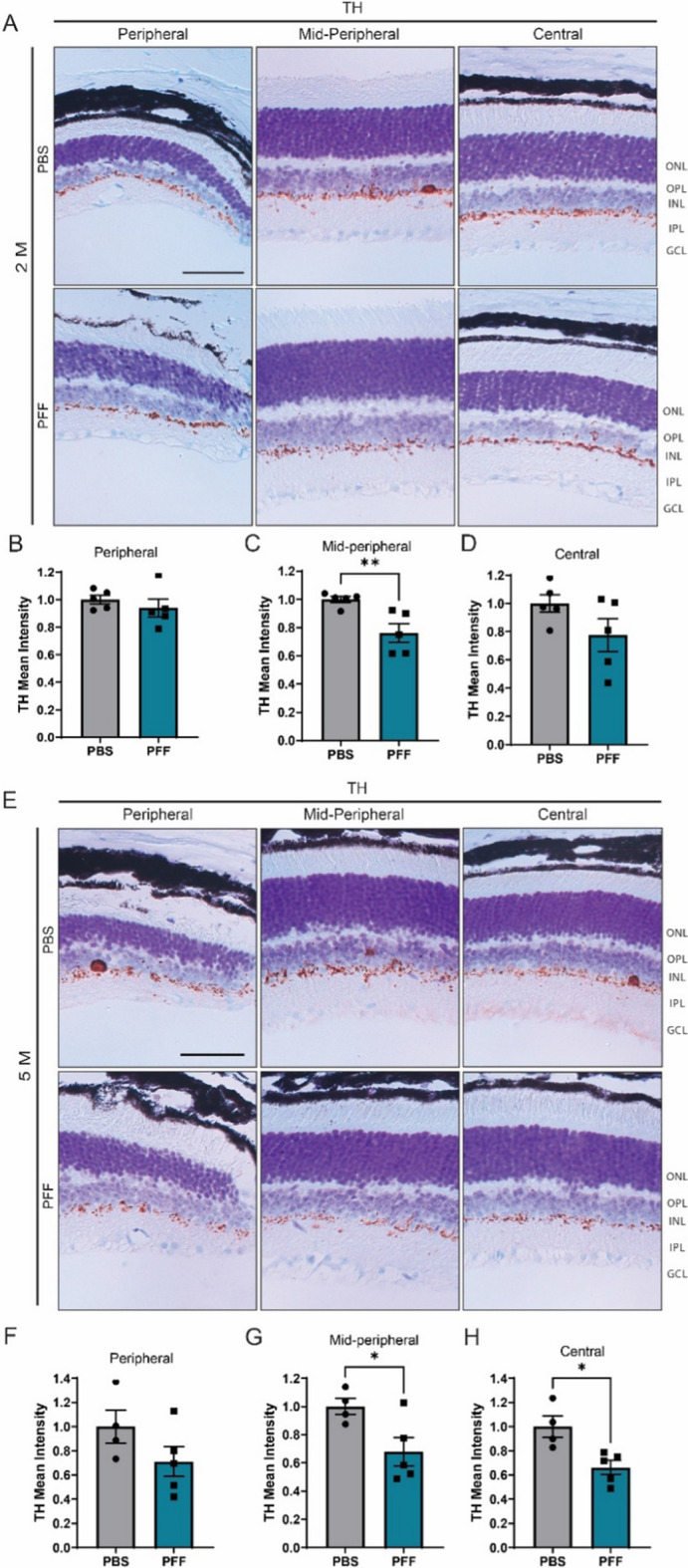
Intravitreal injection of PFFs leads to a decrease in TH levels in the central and midperipheral retina. **A**, **D** Representative images of TH immunostaining in the retina at 2 months (**A**) and 5 months (**E**) after injection. **B**–**D**, **F**–**H** Quantification of the intensity of TH immunoreactivity in the border between the INL and IPL from the peripheral, midperipheral and central retina at 2 (**B**–**D**) and 5 months (**F**–**H**) post injection. Scale bar: 50 µm. Data are expressed as means ± s.e.m, relative to control (PBS, n = 5 mice, PFF n = 5 mice; *p < 0.05, **p < 0.01, ***p < 0.001; Student’s t-test)

The Figures 1 and 5 were updated in this correction article and the original article [1] has been corrected.

## References

[1] Pérez-Acuña, D., Rhee, K.H., Shin, S.J. *et al.* Retina-to-brain spreading of α-synuclein after intravitreal injection of preformed fibrils. *Acta Neuropathol Commun* **11**, 83 (2023). 10.1186/s40478-023-01575-0 10.1186/s40478-023-01575-0 PMC10199563 37210559

