# Supplementary material for: Retina-to-brain spreading of α-synuclein after intravitreal injection of preformed fibrils
Source: Acta Neuropathol Commun. 2023 May 20;11:83. doi: 10.1186/s40478-023-01575-0 (PMC10199563; doi:10.1186/s40478-023-01575-0)
Supplement: Supplementary file 1 — Additional file 1. Supplementary Table 1 (Primer Sequences) and Supplementary Figures 1–4. [file 40478_2023_1575_MOESM1_ESM.docx]

**Supplementary Table 1. RT-qPCR inflammatory cytokine primers.**

| **mRNA** | **Forward primer** | **Reverse primer** |
| --- | --- | --- |
| *IL-1b* | ATCCCAAGCAATACCCAAAGAAGAA | GTGAAGTCAATTATGTCCTGACCAC |
| *TNF-a* | CATCAGTTCTATGGCCCAGACCCT | GCTCCTCCACTTGGTGGTTTGCTA |
| *IFNy* | CTGCTGATGGGAGGAGATGT | TTTGTCATTCGGGTGTAGTCA |
| *IL-10* | TGCACTACCAAAGCCACAAG | TGATCCTCATGCCAGTCAGT |
| *GAPDH* | AGAAGGTGGTGAAGCAGGCATC | CGAAGGTGGAAGAGTGGGAGTTG |


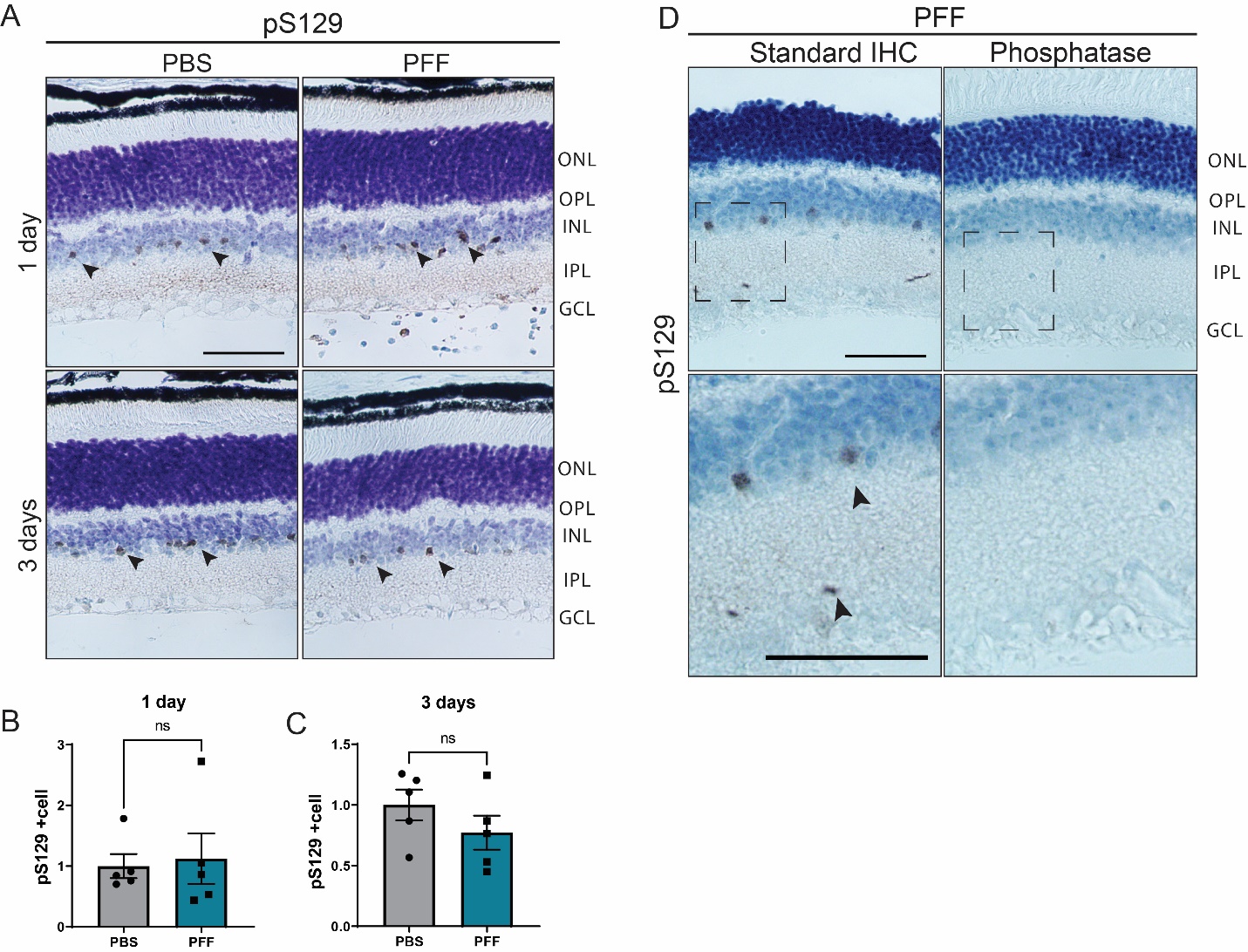


**Supplementary Figure 1. Total α-synuclein levels in the retina after intravitreal injection of PFFs**. (A) Representative images of phospho-α-synuclein inclusions pS129 in the INL of the retina at 1 and 3 days after injection of PFFs. Arrows indicate cell body positive cells (B, C) Quantification of pS129 puncta at 1 and 3 days post injection. (D) Immunohistochemical assessment of pS129 after phosphatase treatment. Representative images of consecutive retinal sections stained with an anti-pS129 antibody. The second section was previously treated with phosphatase. Scale bar: 50 μm. Data are expressed as means ± s.e.m. (PBS, n = 5; PFF, n = 5; *p < 0.05, **p < 0.01, ***p < 0.001; Student’s t-test); ns, not significant (p > 0.05).

**
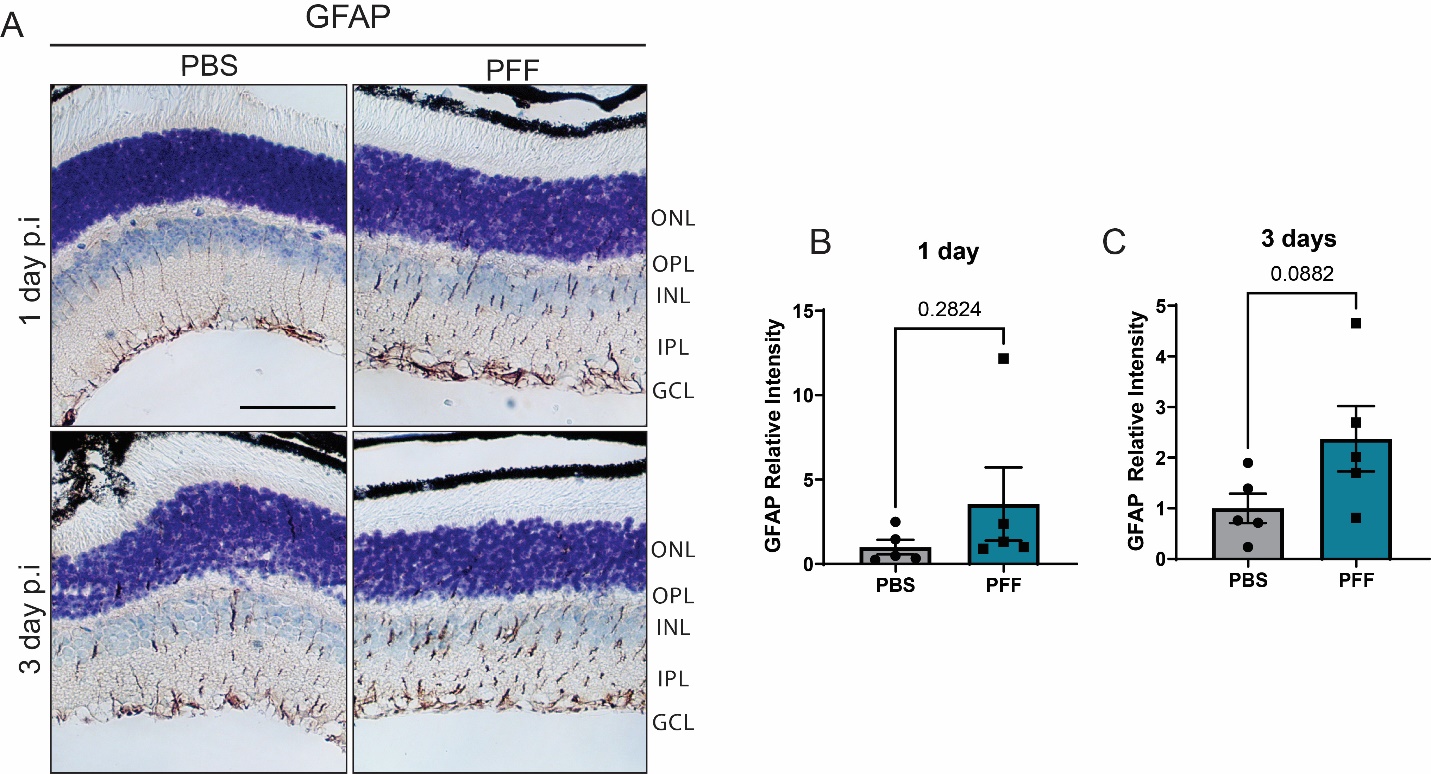
**

**Supplementary Figure 2. Early glial activation following intravitreal injection**. (A) Representative images GFAP in the IPL of the retina at 1 and 3 days after injection of PBS or PFFs. Arrows indicate cell body positive cells (B, C) Quantification of GFAP immunoreactivity at 1 and 3 days post injection. Scale bar: 50 μm. Data are expressed as means ± s.e.m. (PBS, n = 5; PFF, n = 5; *p < 0.05, **p < 0.01, ***p < 0.001; Student’s t-test); ns, not significant (p > 0.05).


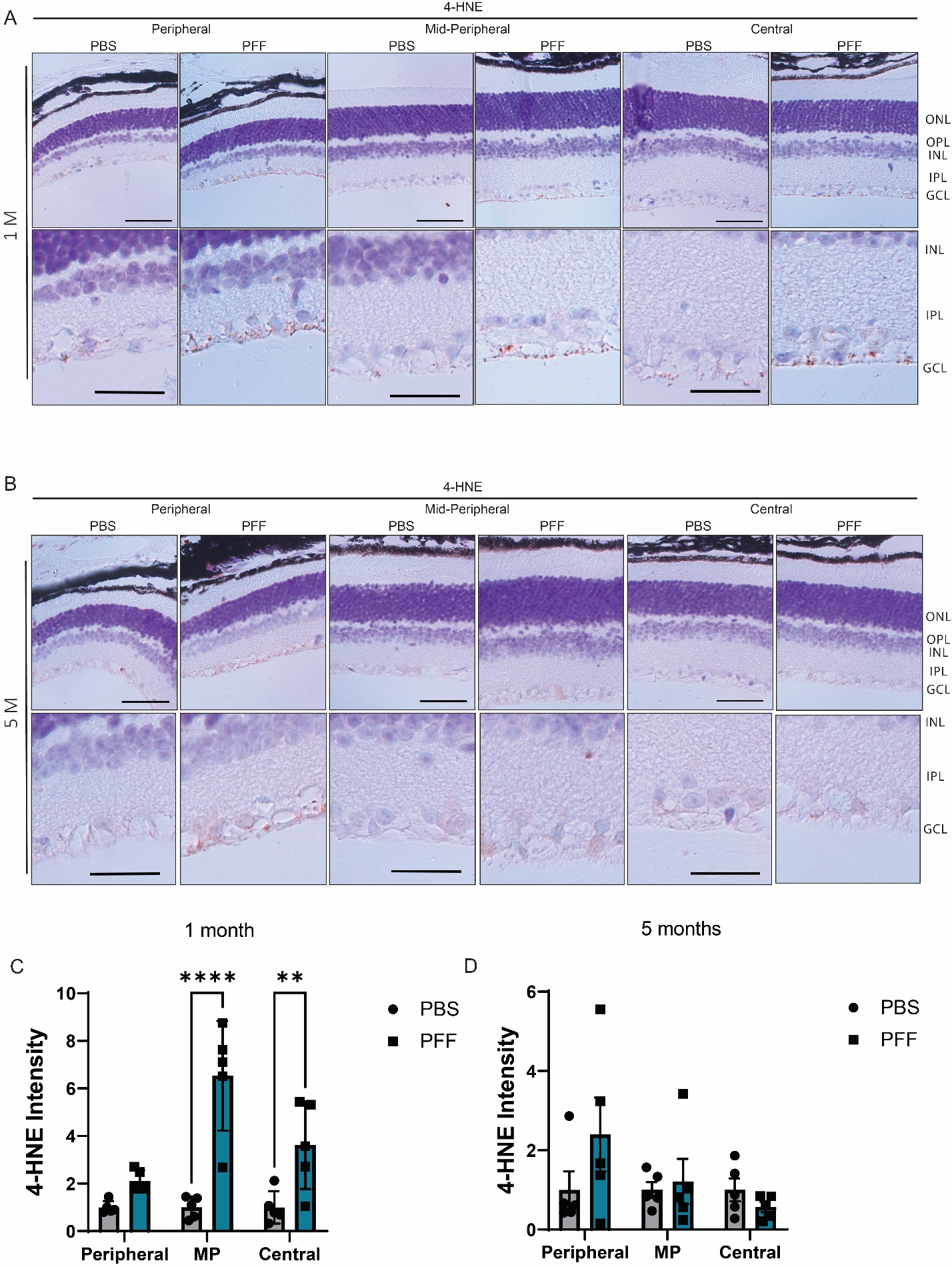

**Supplementary Figure 3. Oxidative stress in the retinal ganglion cell layer at 1 and 5 months after PFF injection** (A-B) Representative images of 4-HNE stained retinal sections at 1 and 5 months after injection of PFFs. Lower panels show higher magnification of GCL (C,D) Quantification of 4-HNE immunoreactivity at 1 and 5 months post injection in peripheral, midperipheral and central retina RGC. Upper panel scale bar: 50 μm, lower panel scale bar: 25 μm Data are expressed as means ± s.e.m, relative to controls (PBS, n = 5–5; PFF, n = 5; *p < 0.05, **p < 0.01, ***p < 0.001; Two-way ANOVA); ns, not significant (p > 0.05).

**
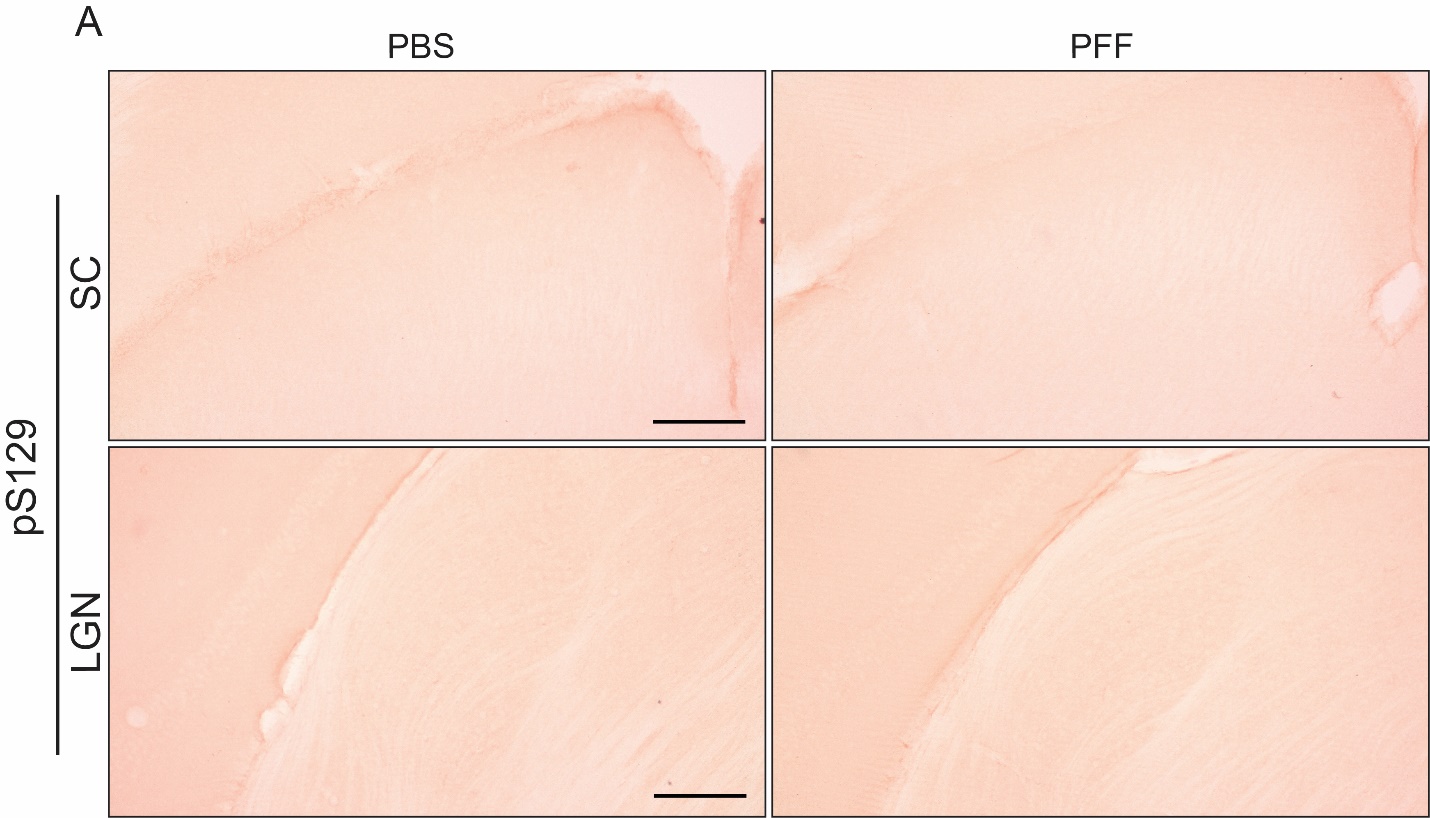
Supplementary Figure 4. Phospho-synuclein staining in the superior colliculus (SC) and lateral geniculate nucleus (LGN)** (A-B) Representative images of pS129 stained SC and LGN brain sections at 5 months after injection of PFFs. No positive pS129 inclusions are observed. Scale bar: 100 μm. (PBS, n = 10 ; PFF, n = 10)
